# Postsurgical Pain and Implant Osseointegration Failure: A Case Control Study

**DOI:** 10.1155/2022/5271892

**Published:** 2022-07-07

**Authors:** Yuli Shang, Qiuying Gao, Tina Lengas, Shu Deng

**Affiliations:** ^1^Department of Stomotology, Tianjin Medical University Second Hospital, 23 Pingjiang Road Hexi District, Tianjin 300211, China; ^2^Boston University Henry M Goldman of Dental Medicine, 635 Albany Street, Boston 02118, Massachusetts, USA

## Abstract

**Aim:**

The relationship between postsurgical pain and osseointegration was evaluated and analyzed in this study. *Material and method*. 27 patients, ranging in age from 35 to 72 years old, 12 males and 15 females, who received dental implants and failed to achieve osseointegration from Tianjin Medical University Second Hospital, were analyzed and studied in the following aspects: bone density, initial torque, one- or two-stage surgery, postsurgical pain, postsurgical swelling, and radiographic evidence of osseointegration failure.

**Result:**

5 patients were assessed to be D4 bone density and 7 cases were assessed to be D3 bone density, 2 patients were assessed to be D2 bone density and 13 patients were assessed to be D1 bone density. All cases were documented with clinically acceptable initial torque. Among the 27 cases, 2 of them were one-stage nonsubmerged surgery and 25 cases were two-stage submerged surgery. 25 out of 27 patients reported moderate to severe pain lasting for more than 72 hours. Radiologic examinations failed to offer any indication of poor osseointegration in the 7-day postsurgical follow-up.

**Conclusion:**

Moderate to severe postsurgical pain lasting more than 72 hours displays high odd ratio of poor osseointegrate. The radiological examinations alone failed to offer any valuable evidence for the early detection of osseointegration failure in this study.

## 1. Introduction

Dental implants have become widely utilized in contemporary dentistry for their efficiency, comfort and reliable, and predictable outcomes [1]. However, dental implant failures could be a challenging concern for both the dental clinician and the dental patient [2]. Dental implant failures impair the patient both physically and mentally and always leave the dental patients and the clinicians under stress [3]. While many studies focus on the etiology of dental implant failure, the mechanism of this complex process still remains unclear [4]. Among various types of dental implant failure cases, failure to achieve osseointegration and the presence of peri implantitis were reported to be the dominant causes among reported unsuccessful dental implant cases [5]. Poor osseointegration could be a catastrophic failure as the dental clinician could take no further steps as long as it occurs. Factors that may impact the osseointegration were reported to be early bacteria contamination [6], poor implant surface design [7], poor surgical delivery skills, and failure to carry out postsurgical management for the patient [8].

Efforts from the manufacturers [9] and the dental clinicians [10] attempting to optimize the ability of implants to osseointegrate are being made consistently. Low bone density has been suspected to be concerning for dental implant osseointegration and many techniques have been developed to optimize the bone condition of the patient [11]. Dental implant design modifications including platform switching could significantly reduce the alveolar bone crest remodeling [12]. Optimization of the implant surface design has also been widely discussed and reported. SLA surface of the dental implants was reported to enhance the surface to be more suitable for early osseointegration [13].

However, poor osseointegration cases continue to be encountered and reported occasionally. Medication including antibiotics and micro molecules still remains no significant beneficial effects when managing poor osseointegrated cases [14].

### 1.1. Objective of the Current Study

Efforts were made in our group to explore the titration of pain control medication application for the dental implant postsurgical patients and several cases of severe postoperative pain that were reported drew our focus and subsequently resulted in a relatively higher possibility of osseointegrated failure encountering in the following up of those cases. Therefore, 27 cases of osseointegrated failure using Dentium Implants were collected and studied in this case-control study to explore any causal relationship between pain and poor osseointegration outcome.

## 2. Material and Method

### 2.1. Experiment Design and Patient Selection

This study was designed to collect 27 patients that encountered dental implant osseointegration failure out of the total 872 patients from the same clinician in Tianjin Medical University Second Hospital between Jan 2018 and Apr 2021. The design and analysis protocols followed STROBE guidelines, and the study was approved by the Ethical Committee of the Second Hospital of Tianjin Medical University.

Patients who participated in this study displayed no detectable contraindications of receiving the dental implants, and all the consent forms and necessary blood work had been accomplished prior to the dental implant installation procedure. Excluding criteria include as follows: younger than 18 years of age; inability to fully understand or report the NRS reporting system; females using oral contraceptives, being pregnant or actively breastfeeding; chronic use of bisphosphonates within 3 years prior to the study; uncontrolled periodontitis, TMD, and untreated caries.

### 2.2. Control Group Setting

30 cases of patients with successful osseointegration (age range from 27 to 71, 14 males and 16 females, 6 two stages and 24 one stages) were randomly selected and evaluated as the control group and 6 patients among the 30 cases received a bone graft (to reduce the selection bias).

### 2.3. Clinical Examination Parameters

All the patients received the Dentium Superline implants following a standard protocol by the same clinician from Tianjin Medical University Second Hospital. All the patients were followed up by phone on the 1st and 3rd days post implant surgery. Sutures were removed on the 7th day post surgery, and the nonsubmerged case patients were also called back for clinical evaluation and X-ray examination. In case of abnormal issues reported, all the abnormal information including nonrelieved moderate to severe pain or severe localized swelling were clearly documented and one additional in-clinic appointment was scheduled on the 14th day post surgery for those patients.

### 2.4. NRS Reports and Data Collection

All the data from the dental implants osseointegration failure patients including the following aspects were collected and analyzed: bone density classification, initial torque, one- or two-stage surgery, postsurgical pain, postsurgical swelling and radiology indication and fixture removing time. Pain classification was collected and measured by using a questionnaire with the pain classification numerical rating scales (NRSs), and mild pain was defined with a scale of 1–3, moderate was defined as a scale of 4–6, and a severer pain was defined as more than 7 in this study [15, 16].

### 2.5. NRS Report Calibration and Sensitivity Test

All the patients were checked to report the pain score number describing the pain for blood taking procedure in order to test the bias of NRS evaluation. 12 patients out of 27 osseointegration failure patients and 9 out of the 30 control patients reported a dental history of acute pulpitis and the number was confirmed to evaluate the sensitivity of the NRS pain report.

### 2.6. Investigator-Blinded Radiology Examination Analysis

All the radiologic examinations from the 27 patients in which the dental implants failed to osseointegrate and the 30 patients from the control group were collected and evaluated by an independent experienced dental clinician by a blind design method to detect any suspected indication of osseointegration failure.

### 2.7. Statistical Analysis

Data collected were analyzed by using SPSS20.0, and all the data were displayed with mean and 95% confidence intervals. Comparisons were performed and analyzed between the osseointegration failure group and the control group by the comparative *t*-test after checking the distribution of the data and *p* < 0.05 was defined as significantly different in this study.

## 3. Result

### 3.1. Evaluation of the Overall Success Rate of Dentium Superline Implant Osseointegration

Analyzing all the patients who received Dentium Superline implants delivered by the clinician involved in this study, 1426 Dentium Superline implants were delivered to 872 patients from Tianjin Medical University Second Hospital between Jan 2018 and Apr 2021, and 27 out of 1426 Dentium Superline implants failed to achieve osseointegration. The overall implant osseointegrated cumulative success rate is 98.1%.

### 3.2. D1 Bone Density Accounts for More than Half of the Osseointegration Failure Cases

Among the 27 patients encountering dental implant osseointegrated failure, 13 of them were clinically accessed to be D1 bone density, 2 patients to be D2 bone density, 7 patients to be D3 bone density, and 5 patients to be D4 bone density. The results are shown in Table 1.

### 3.3. Torque Control for the Placement of the Osseointegration Failure Implants

**Figure 1:**
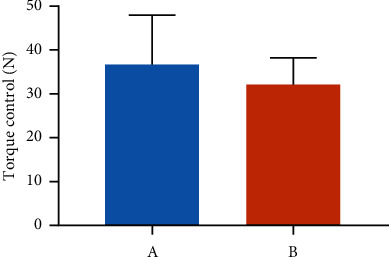
Torque control for the installation of implants in two different groups. A: osseointegrate failure (OF) group; B: control group.

Based on the documentation of all 27 patients, the initial torque during placement of the Dentium Implants for the 27 patients was controlled between 20 N·cm to 60 N·cm with an average of 36.67 N·cm (95% CI: 32.2–41.2). This result showed no significant differences when compared with the control group 32.17 N·cm (95% CI: 30.35–36.65). The results are displayed in Figure 1.

### 3.4. High Chance of Moderate to Severe Postsurgical Pain Lasting for More than 72 Hours Was Reported by the Osseointegration Failure Group

The average NRS number for blood taking from osseointegrated failure group was 2.12 (95% CI: 1.77–2.46) and the NRS reported from the control group is 2.45 (95% CI: 2.11–2.78), and there were no significant differences between the two groups (*p*=0.16). The average NRS number reported for severe pulpitis is 7.75 (95% CI: 7.14–8.36) from the osseointegration failure group and 7.78 (95% CI: 6.94–8.62) from the control group, and no significant differences were found (*p*=0.95).

**Figure 2:**
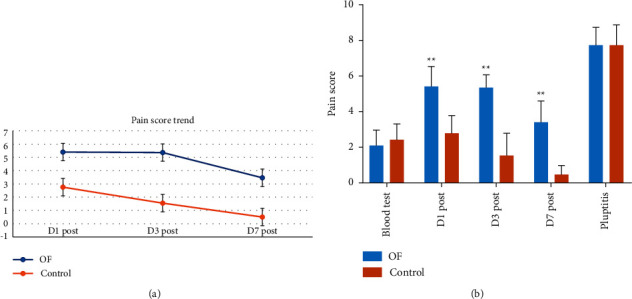
(a) Postsurgery pain score trend in two different groups. (b) Pain score distributions reported by the patients under different following-up time points in two groups (^*∗∗*^*p* < 0.01, versus of group and control group).

The average 1-, 3-, and 7-day(s) postsurgery pain reported by the 27 osseointegration failure patients were 5.42 (95% CI: 4.97–5.86), 5.35 (95% CI: 5.05–5.65), and 3.46 (95% CI: 3.02–3.91), respectively, which is significantly higher than those of the control group, which were 2.76 (95% CI: 2.37–3.15), 1.55 (95% CI: 1.07–2.03), and 0.483 (95% CI: 0.289–0.676). The results are displayed in Figure 2(a). The distribution of the pain reported by the patients under different post-surgery following up time is visualized in Figure 2(b).

### 3.5. Two-Stage Surgery Displayed a Higher Odds Ratio of Osseointegration Failure

25 cases out of 27 dental implants osseointegration failure cases received the Dentium Superline implant with a two-stage submerged surgery and 2 out of 27 patients went through the one-stage nonsubmerged procedures. The results were displayed in Table 2 and the odds ratio of encountering osseointegration failure is 60.85 when compared two stages surgery with one-stage surgery in this study.

### 3.6. 88.88% of the Overall 872 Patients Reported No Pain or Mild Pain within 7 Days Post Surgery

547 patients reported mild pain with no intervention with any pain control medication. 97 patients reported by phone follow-up, moderate to severe pain on the 1st day post surgery, 89 of them continued to report moderate to severe pain on the 3rd day post surgery phone follow-up, and 94 of them were administrated with NSAID (Motrin 600 mg Q6 h prn pain) or acetaminophen (650 mg Q6 h prn pain) after ruling out contraindications. All the 97 patients were scheduled for an additional appointment on the 14th day post surgery. 42 patients still complained of pain on the 7th day post surgery when removing suture or in clinic recall and 25 out of 42 patients encountered dental implant osseointegration failure. The other 2 patients who encountered osseointegration failure reported mild to moderate pain on the 1st and 3rd day but lasting no longer than 5 days. The odds ratio of poor osseointegration between the longer than 72 hours moderate to severe pain patients and no pain or mild pain patients was 153. The results are displayed in Table 2.

### 3.7. Overall 617 Patients Out of 872 Patients Reported Localized Swelling within the First 5 Days Post Surgery with a Peak in Swelling at Day 3 Post Surgery

The local swelling was followed up on the 1st and 3rd day post surgery by phone and examined by the clinician on the 7-day follow-up. 617 out of 872 patients reported swelling and the swelling peak occurred on the 3rd day postoperative follow-up. 150 out of the 617 patients reported long-duration swelling on the 7th day post surgery following up and all of them were scheduled to have an additional follow-up on the 14th day post surgery. Unfortunately, among the 150 patients, 19 were found encountering poor osseointegration 1 or 2 weeks later. The odd ratio of poor osseointegration between longer than 7 days swelling patients and none or mild swelling patients is 12.94. The results are shown in Table 2.

### 3.8. Radiology Examination on 7 Days Post Surgery Follow-Up Failed to Offer Any Alarming Indication for the Early Poor Osseointegration

**Figure 3:**
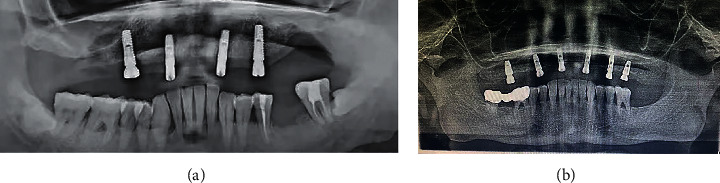
(a) One of the 14th-day postsurgery panoramic examinations. The maxillary right implants failed and were removed in the following weeks. There is no clue detectable for the dental clinician at the current time. (b) The maxillary right premolar region implant was suspected to encounter poor osseointegration when being reviewed on the 7th day and 14th day post surgery; however, it achieved osseointegration in the following weeks and still functions very well in the patient's mouth for the time being.

3 out of 27 (osseointegration failure group) and 3 of 30 (control group) were suspected by the independent dental clinician when reviewing the 7th-day postsurgery X-ray. On the 14^th^-day postsurgical appointment, 16 out of 26 patients (osseointegration failure group) were radiology suspected for poor osseointegration. One panoramic from one osseointegration failure patient and one panoramic from a successfully osseointegrated patient typically displayed the misleading information acquired from the radiology examination in Figures 3(a) and 3(b), respectively.

All the abbreviations used in this study are collected and displayed in Table 3.

## 4. Discussion

Failing to achieve osseointegration tends to be very challenging for dental implant treatment as there are very limited interventions that can be implemented to combat this process [17].

The overall osseointegration failure rate in the current study was 1.83%, which is consistent with the reports of relevant literature [18]. Despite the overall failure rate being relative low, the impact of dental implants osseointegration failure could be destructive and costly. In the current study, all the patients lost 20–40% of their buccal bone volume after 1 month of the implant removal procedure.

Methods aiming to lower the risk of poor osseointegration encountering have been widely studied and reported by manufacturers and dental clinicians. In general, the methods include surface modification [19], design optimization [9] and the protocol upgrading of dental implant installation [20]. The contour of the implants is still being explored by dental clinicians and manufacturers as the contour would play a key role in the stress distribution when placing the implants, which could only be roughly measured as the initial torque in the dental clinic [21]. In this current study, the bacterial cultures were carried out after the patient encountering poor osseointegration, as bacteria contamination was highly suspected to be relevant to the inflammatory status of the bone [22]; however, the bacteria culture data failed to give any significant differences in this study. The installation torque values were collected and analyzed and the findings from the current study tend to prove that extra torque during installing the implants may increase the risk of osseointegration failure, which is consistent with the findings of Irinakis and Wiebe [23]. In addition, excess torque may diminish the blood supply around the adjacent tissue of the implant [24] and disturb the blood blot formation, this could be a potential aspect to be explored in the future study.

There are several clinical decisions including preventing surface contamination and postsurgical management to be taken into account when managing dental implant treatment [25]. Detecting the early onset of poor osseointegration could reasonably prevent the patient from losing valuable bone as well as a considerable chance of unnecessary pain [26]. It would be beneficial for the dental clinician to establish a follow-up protocol during the postsurgical period and individualize the protocol accordingly.

Based on the NRS data from the current study, moderate to severe postsurgery pain lasting for more than 72 hours could be an early indicator of the early lack of implant osseointegration. Attempting to address the causal association between long-lasting moderate to severe postsurgical pain and poor dental osseointegration, one of the hypotheses discussed in our group was that the inflammatory secretions elevate the tension of the periosteum and simulate the free nerve endings continuously and this hypothesis is consistent with the study reported by Aysan Shahnaz et al. [27]. Additionally, during the study, 5 severe postsurgery pain patients received a second-stage surgery 1 or 2 days after reporting severe pain and all of them reported significant pain relief 1 or 2 days after the healing cap insertion. This result could partially address that applying a healing cap potentially helped drain the exudate from the soft tissue and the bone socket around the implants, and this could potentially explain the different outcomes between one-stage surgery and two-stage surgery in this study.

The dental clinician from the current study reported no preference to take one-stage or two-stage surgery for the patients, except for bone graft cases or failing to match an ideal healing cap for the patients. However, as bone grafts were carried out for 5 of the osseointegration failure cases and 49 cases of the successful osseointegrated patients, the influences from the bone grafts were not independently analyzed in the current study. As the bone graft cases were all carried by a two stages submerged surgery, the selection bias from the current study may not be ideally minimized. The surgical size, site, and flap management may influence the pain severity reported by the patients; however, in more than 10 cases of all-on-six procedures, the postsurgery pain will be dramatically relieved within 48 hours post surgery in the current study. The blood test NRS reported by patients from the osseointegration failure group and control group was evaluated to minimize the reporting bias in the current study. The acute pulpitis NRS data was utilized to test the sensitivity of NRS in the current study and based on the result, all the patients displayed reliable reporting tendencies. One issue that should be taken into account is post-dental treatment pain could be TMJ-related. Based on this point of view, this part of bias could not be totally ruled out in this study as the pain reported by the patient could be of TMD origin rather than the implant surgery origin [28].

Titration of postimplant pain control medication explored in Tianjin Medical University Second Hospital before the current study was carried out, and some cases of moderate to severe pain were reported occasionally. Patients who received dental implants would be routinely prescribed NSAID or acetaminophen post surgery when ruling out all the contraindications, which is consistent with the current protocol [29]. In the current study, no opioid analgesics are getting involved due to the regulations for the dental procedure of opioid analgesic medication application in China. Even though opioid analgesic medication could be a popular choice to prevent the patient from suffering moderate to severe post-dental procedure pain in the US and many European countries [30], there is very limited amount of opioid analgesic medication applied in post-dental procedure pain control in China for the time being. The efficacy evaluations of NSAIDs and acetaminophen were roughly carried out in the current study, but the results failed to offer any valuable information.

In the current study, the D1 bone density displayed a higher odd ratio of osseointegration failure. Based on the documentation of the patients in the current study, additional osteotomy efforts would always be made to load the implant into the ideal depth. This may be concerning as the more time and effort the osteotomy takes, the more thermal trauma might potentially impact the bone [31]. In addition, the limited blood supply due to a limited amount of the spongy bone in the D1 bone could also be concerning as failing to fill the osteotomy socket with blood may lead to insufficient cells and nutrition for the new bone formation, leaving the osseointegration poor outcome [32]. In general, many procedures have been established to load the implant with an optimal torque and ideally, the D2 and D3 bone density patient is a better candidate for implant installation [11].

The radiological examination acts as a vital technique in making a dental diagnosis, treatment plan, or following up [33]. In the current study, bite wing, PA, panoramic, and CBCT all failed to rule out or confirm the poor osseointegration on the 7th day postsurgery follow-up. There were some detectable clues found on the 14th day postsurgery follow-up; however, for many dental clinics, there are no routine 14-day post-dental implant surgery follow-ups. Considering that many dental clinicians tend to schedule the patients for 3 to 4 months post surgery for the second-stage surgery appointment [34], this long interval may leave the patients and the dental clinicians at risk. Based on our study, utilizing teledentistry in the postsurgical management for the patient might be beneficial for early detection of abnormal osseointegration status, buying the dental clinician and patient valuable time to intervent and decrease the risk of losing bone, unnecessary pain, and the possibility of maxillofacial infection. This is consistent with literature from Minervini et al. [8]. Therefore, systemically following up on the patients and evaluating the patient's pain reports could act as an alternative protocol to upgrade the post-dental implant patient management.

Dental implant-related pain has been reported frequently and many dental clinicians are paying attention to this new aspect. Pain encountering could be reported at any stage during the implant treatment, including post implant installation and post prosthetic procedure. Many innovative techniques including telescopic dentistry utilized in the prosthetic procedure for the patients are available, making the prosthetic procedure more predictable [35]. However, we still suspect that early detection of prosthetic-related pain could facilitate the dental clinician to carry problem shooting in an early stage. Therefore, future studies are needed to be carried out in exploring the role of postprosthetic pain in dental implant maintenance. In addition, innovative medications including microRNA localized injection [14] or biomaterial products such as collagen frame carrier [36] needed to be invented or upgraded in the future as a predictable intervention is eagerly needed to turn over the poor osseointegration once it occurred.

More well-designed random control trial studies are needed to confirm the causal relationship between severe pain and osseointegration failure and future studies would also be needed to explore the physiological mechanism of the outcome in this study.

## 5. Conclusion

Moderate to severe pain post dental implant surgery lasting more than 72 hours displayed a higher odds ratio of osseointegration failure. The radiographic examination failed to offer any predictable information on the early detection of poor implant osseointegration in this study. As a case-control study, we are looking forward to seeing more well-designed random controlled trial studies to be carried out to confirm the casual relationship between postsurgical pain and implant osseointegration failure. Also, the mechanism of the outcome of the current study needs more evidence to explain.

## Figures and Tables

**Table 1:** Bone density distribution of osseointegrated failure patients.

Bone density	D1	D2	D3	D4
Pat. number	13	2	7	5

**Table 2:** Patient distribution with different relative factors.

		Osseointegration failure	Osseointegrate success
Surgical procedure	Two-stage submerged	25	144
	One-stage nonsubmerged	2	701

Postsurgical pain	Moderate to severe pain, last >72 h	25	64
	No more than mild pain, last <72 h	2	781

Postsurgical swelling	Moderate to severe swelling, last >5 d	19	131
	No more than mild swelling, last <5 d	8	714

**Table 3:** Abbreviations.

Abbreviation	Full name
SLA	Sand blasting, large grit, acid-etched
NRS	Numerical rating scales
TMD	Temporomandibular disorder
CI	Credibility interval
NSAID	Nonsteroidal anti-inflammatory drug
PA	Peri apical (X-ray)
CBCT	Cone beam computed tomography

## Data Availability

The data for this study were collected from patients' medical records in Tianjin Medical University Second Hospital. The patient recording data used to support the findings of this study are restricted by the Ethical Community of Tianjin Medical University Second Hospital in order to protect patient privacy. The data are available from dengshu1988@hotmail.com for researchers who meet the criteria for access to confidential data.
